# Pilot observational study on haemodynamic changes after surfactant administration in preterm newborns with respiratory distress syndrome

**DOI:** 10.1186/1824-7288-40-26

**Published:** 2014-03-05

**Authors:** Francesca Vitali, Silvia Galletti, Arianna Aceti, Giulia Aquilano, Marianna Fabi, Anna Balducci, Giacomo Faldella

**Affiliations:** 1 Neonatology and Neonatal Intensive Care Unit, S. Orsola-Malpighi Hospital, University of Bologna, Bologna, Italy; 2 Pediatric Cardiology and Cardiac Surgery, S. Orsola-Malpighi Hospital, University of Bologna, Bologna, Italy

**Keywords:** Echocardiography, Preterm infant, Surfactant, Tissue doppler imaging, Tricuspidal annular plane systolic excursion

## Abstract

**Background:**

Surfactant treatment reduces respiratory morbidity and mortality in preterm infants. Data on its haemodynamic consequences are conflicting. The aim was to characterise the haemodynamic effects of surfactant treatment on cardiac function in preterm newborns with respiratory distress syndrome (RDS).

**Methods:**

Preterm infants (gestational age <34 weeks, birth weight <2000 g) with RDS, who received surfactant within 72 hours of life, were recruited.

Echocardiography was performed before surfactant, and 2 and 24 hours after. Left and right ventricular peak systolic, early diastolic and late diastolic myocardial velocities were measured using Tissue Doppler Imaging (TDI), while characteristics of the *ductus arteriosus*, pulmonary artery pressure, right ventricular (RVO) and left ventricular output were measured by standard echocardiography. Tricuspidal Annular Plane Systolic Excursion (TAPSE) was measured on the free wall of the tricuspid annulus.

**Results:**

Fourteen patients were studied. Surfactant was associated with a decrease in pulmonary pressure and an increase in RVO. The improvement of right ventricular function was also confirmed by a significant increase in right peak systolic velocity and in TAPSE. Left ventricular velocities did not change significantly after surfactant.

**Conclusions:**

Surfactant administration in preterm infants with RDS did not impair myocardial contractility and was followed by increased RVO, in agreement with other parameters of right ventricular function. TDI and TAPSE appeared to be reliable and feasible in this population. The addition of TDI and TAPSE to standard neonatal echocardiography may provide additional information about cardiac function.

## Background

Respiratory distress syndrome (RDS) represents one of the major causes of neonatal morbidity and mortality in preterm infants [1]. Moderate and even severe RDS are increasingly being treated with nCPAP and surfactant administration using the “InSurE” procedure (Intubation, Surfactant, Extubation) rather than with mechanical ventilation [2]. Treatment with surfactant improves oxygenation and lung compliance [3]. The reduction in pulmonary vascular resistance following surfactant administration may increase pulmonary blood flow [4-6]. In the presence of a patent *ductus arteriosus* (PDA) and a large systemic-to-pulmonary pressure gradient, systemic blood flow and end-organ perfusion may fall substantially [4-9]. Data on the haemodynamic consequences of surfactant administration are conflicting [10-12]. It has been shown that surfactant administration can be associated with changes in brain haemodynamics, which may contribute to the development of intracranial haemorrhage and periventricular leukomalacia, but the mechanism by which this occurs is still unclear [10,13]. Furthermore, it has been shown in previous studies that surfactant administration is associated with a low diastolic arterial pressure; the consequences of this change is unknown, but possible effects include compromised coronary artery perfusion leading to myocardial fatigue and low cardiac output [10].

Previous studies have investigated cardiac function after surfactant therapy in preterm infants using standard ultrasound methods. These methods have several limitations in preterm newborns, because cardiac function is affected by heart rate, preload and afterload, which are extremely variable during the first days of life. Pulmonary artery pressure (PAP) can also be assessed non-invasively using Doppler echocardiography [14]. Tissue Doppler Imaging (TDI) is a relatively new echocardiographic technique which provides velocities of the myocardial wall during the cardiac cycle; it allows the quantification of both regional and global systolic and diastolic function and has the advantage over pulse-wave Doppler (PWD) of being less dependent on preload and afterload than standard echocardiography (ECHO) [12,15-18].

The Tricuspid Annular Plane Systolic Excursion (TAPSE) is an echocardiographic measurement which assesses right ventricular systolic function in a simple, repeatable, and highly reproducible way. Until recently, TAPSE was not part of routine echocardiographic assessment in neonatal care: normal values for preterm and term neonates, with respect to gestational age and birth weight, have been proposed only in 2011 [19].

An improved understanding of the haemodynamic consequences of surfactant administration using new echocardiographic techniques may further enhance our clinical approach to the preterm population.

For this reason, the aim of the present study was to characterise the haemodynamic effects of surfactant treatment on cardiac function in preterm newborns with respiratory distress syndrome (RDS), comparing standard ultrasound methods with ECHO measures recently applied to assess cardiac function in preterm infants, such as TAPSE and TDI.

## Methods

### Study population

This prospective, pilot study was conducted between December 2011 and May 2012. Preterm infants consecutively admitted to the Neonatal Intensive Care Unit (NICU) at S.Orsola-Malpighi Hospital - Bologna, who satisfied the following criteria were included in the study:

– Birth weight <2000 g;

– Gestational age 26-34 weeks;

– Moderate to severe RDS, treated with selective endotracheal surfactant replacement with the InSurE procedure within 72 hours of life.

Exclusion criteria were the following:

– Congenital heart disease (CHD), other than PDA and patent *foramen ovale* (PFO);

– Major congenital malformations;

– Sepsis;

– Need for mechanical ventilation.

### Study design

According to the guidelines for the management of RDS in our NICU, on admission all the infants received non-invasive respiratory support with nCPAP. They were then intubated, given surfactant and extubated. Two-dimensional ECHO was performed before surfactant administration (T0), and 2 (T1) and 24 hours (T2) after. Timing of surfactant administration ranged from 1 to 72 hours of life.

The study protocol was approved by the Institutional Ethics Committee of S.Orsola-Malpighi Hospital, Bologna. Written informed consent was obtained from each patient parent/guardian.

### InSurE procedure

Before intubation, infants received a single i.v. bolus of fentanyl (1 mcg/kg) and, when necessary, they were briefly ventilated with bag and mask and additional oxygen to maintain SpO2 values between 85 and 94%. After intubation, surfactant (Curosurf 200 mg/kg; Chiesi Farmaceutici SpA, Parma, Italy) was given endotracheally, and the administration was followed by manual ventilation through the endotracheal tube for 2-5 minutes. When good respiratory drive and satisfactory transcutaneous oxygen saturation (SpO2 ≥ 85% with FiO2 ≤ 30%) values were established, infants were extubated and nCPAP was restarted using a CPAP level equal or 0.5 cmH_2_O lower than the one required before the procedure.

### Echocardiographic measurements

The ECHOs were performed using a Philips HD11XE System (Philips Ultrasound, Andover, MA, USA) with a S12-4 Hz transducer. A single operator performed all the ECHO scans. Post processing analysis was performed after the scan.

Using ECHO, ductal characteristics, pulmonary artery pressure, mitral flow velocity, ventricular output for both ventricles and myocardial wall velocities were evaluated. CHD other than PDA or PFO were excluded by the T0 scan.

All measurements were taken repeatedly over three to five consecutive cardiac cycles to minimise variations related to the respiratory cycle; the mean value for each ECHO measurement was used for the analysis. For Doppler evaluations, images were optimised to ensure that the angle of insonation was approximated to ‘zero’, in order to minimise flow underestimation.

#### Standard ECHO

The *ductus arteriosus* was evaluated from a suprasternal notch view using a combination of two-dimensional and colour Doppler echocardiography [20]. If PDA was present, the transductal diameter was calculated with two dimensional ECHO. Then, the pulsed Doppler gate was carefully placed in the pulmonary end of the *ductus* and the flow pattern of the shunt was recorded. Three Doppler patterns were identified: pulsatile or unrestrictive, bidirectional and restrictive.

Using a parasternal long axis view, 2D M-mode measurements were performed to obtain the left atrium to aortic root ratio (LA:Ao). TAPSE was measured using an apical four chamber view with the cursor placed on the free wall of the tricuspid annulus [21]. Maximal TAPSE was determined by the total excursion of the tricuspid annulus from its highest position after atrial ascent to the lowest point of descent during ventricular systole.

From the same view, mitral inflow velocities were also obtained by a PWD sample gate at the valve leaflet tip. Offline analysis was used to calculate early diastolic flow velocity (E), late diastolic flow velocity (A) and their ratio (E/A).

Left ventricular output (LVO) was calculated according to the formula [(left ventricular outflow velocity time integral) [VTI] × (heart rate) × (left ventricular outflow cross-sectional area)], and indexed to body weight. The left ventricular outflow diameter was measured using a parasternal long axis view using the leading edge technique [22]. VTI was estimated from an apical five-chamber view with the sample volume placed in the left ventricular outflow tract.

Right ventricular output (RVO) was evaluated from an oblique long axis view and calculated according to the formula [(right ventricular outflow VTI) × (heart rate) × (right ventricular outflow cross-sectional area)], indexed to body weight. Both left and right VTIs were calculated using the average of five consecutive cardiac cycles to minimise variations related to the respiratory cycle [22].

Pulmonary artery pressure (PAP) was calculated using the ratio of the acceleration time (AT) to right ventricular ejection time (RVET). AT/RVET correlates inversely with PAP [14]. AT refers to the time interval from the beginning of ventricular systole to the achievement of peak velocity, while ET to the time interval from the beginning to the end of ventricular systole. AT and RVET measurements were made with the Doppler velocity waveform. The values above were obtained with a minimum of five consecutive waveforms. Because mean AT/RVET did not correlate significantly with the R–R interval at the ECG, we decided to not perform correction of AT/RVET for the heart rate as described in other papers [23,24].

#### TDI analysis

Myocardial velocities were obtained using an apical four-chamber view and were measured at the basal segments. Pulsed-wave TDI was performed by adjusting the spectral pulsed Doppler signal filters to obtain a Nyquist limit of 15–20 cm/s. High-frame-rate (150 frames/s) images were acquired in the tissue Doppler mode. A PWD sample gate of 0.12 cm was positioned at the lateral tricuspid and mitral annuli (LTA and LMA, respectively) to acquire right and left ventricular velocities. Peak systolic (S’), early diastolic (E’) and late diastolic (A’) myocardial velocities were measured for each ventricle. E/E’ ratio was calculated for the left ventricle.

### Statistical analysis

Data were analysed using SPSS 16.0 for Windows (Statistical Package for Social Sciences, SPSS Inc., Chicago, Ill, US). Data were not normally distributed, thus non parametric statistical methods were used. Differences between ECHO measures taken at T0, T1 and T2 were evaluated by Wilcoxon Signed Ranks Test. The relationship between left ventricle E and E’ at T0, T1 and T2 were evaluated by Spearman Test. A *p* value < 0.05 was considered as statistically significant.

Similarly to previous studies [16], intra-observer reproducibility was evaluated by calculating coefficient of variations (CVs), expressed as (standard deviation/arithmetic mean of measurements) × 100.

## Results

### Clinical data

The characteristics of the patients are detailed in Table 1. Over the study period, all the infants had normal heart rate (range, 125-168 bpm) and normal blood pressure; none of them required inotrope support.

**Table 1:** Characteristics of the patients included in the study

Gender (M/F)	11/3
Congenital heart disease	0/14
Gestational age (weeks); median, IQR	30, 27.5-33.4
Birth weight (grams); median, IQR	1162, 500-1990
nCPAP requirement on admission	14/14
Pressure (cmH2O); median, range	4, 3.5-5

#### Standard ECHO data

The characteristics of PDA at T0 are detailed in Table 2. At T1, mean TDD and transductal blood flow did not change significantly. At T1, a non-significant increase in LA:Ao was detected, compared to T0 (*p* = 0.085).

**Table 2:** Characteristics of patent ductus arteriosus

Presence of patent ductus arteriosus @ T0	14/14
Transductal diameter @ T0 (mm); mean, SD	2.1, 0.3
Exclusive left-to-right transductal blood flow @ T0	8/14
Presence of right-to-left transductal blood flow @ T0	6/14
Proportion of right-to-left transductal blood flow @ T0; median, range	25.5%, 24-28

At T2, transductal blood flow was exclusively left to right in 9/14 patients. *Ductus arteriosus* was found to be closed in 2/14 infants. In the remaining 3 patients, ductal blood flow had not changed.

Echocardiographic data are reported in Table 3. No difference was detected in transmitralic E and A at T1 and T2, compared to T0. There was no change in transmitralic E/A ratios over time. In 13 patients the AT/RVET ratio increased significantly at T2 compared to T0. There was no increase in the AT/RVET ratio only in the patient who developed a pneumothorax after surfactant administration.

**Table 3:** Echocardiographic parameters measured before the administration of surfactant (T0), and 2 hours (T1) and 24 hours (T2) after

	**T0**	**T1**	**T2**	***p value***	***p value***	***p value***
				**T0 *vs *** **T1**	**T0 *vs *** **T2**	**T1 *vs *** **T2**
AT/RVET	0.25 ± 0.05	0.30 ± 0.06	0.33 ± 0.06	0.066	**0.001**	**0.011**
Left ventricle						
E (cm/sec)	40.84 ± 11.84	40.24 ± 4.63	45.85 ± 15.56	0.953	0.272	0.374
A (cm/sec)	51.13 ± 14.98	49.47 ± 7.79	52.12 ± 14.2	0.260	0.925	0.260
E/A	0.81 ± 0.13	0.83 ± 0.13	0.88 ± 0.17	0.192	0.300	0.314
LVO (ml/Kg/min)	204.13 ± 65.24	210.89 ± 76.42	227.91 ± 77.41	0.779	**0.041**	0.327
RVO (ml/Kg/min)	220.82 ± 95.57	265.08 ± 65.88	256.86 ± 113.09	0.674	**0.050**	0.889
Left ventricle						
S’	3.26 ± 0.51	3.50 ± 0.64	3.52 ± 0.67	0.085	0.372	0.953
E’	4.75 ± 1.01	4.59 ± 0.77	5.02 ± 1.54	0.406	0.593	0.086
A’	4.16 ± 1.13	4.56 ± 1.57	5.05 ± 2.04	0.314	0.124	0.260
Right ventricle						
S’	4.64 ± 0.75	4.60 ± 1.05	5.27 ± 0.87	0.859	**0.035**	0.260
E’	5.47 ± 1.12	5.67 ± 1.12	6.24 ± 1.69	0.214	0.198	0.515
A’	6.88 ± 1.37	7.08 ± 1.78	7.48 ± 0.97	0.678	0.272	0.374
Left ventricle						
E/E’	8.77 ± 2.46	8.98 ± 1.60	9.48 ± 3.06	0.953	0.509	0.767
TAPSE (cm)	0.72 ± 0.17	0.73 ± 0.12	0.85 ± 0.13	0.767	**0.002**	**0.008**

Data are presented as mean ± SD. Significant *p* values are reported in bold.

AT: acceleration time; RVET: right ventricular ejection time; LVO: left ventricular output; RVO: right ventricular output; TAPSE: Tricuspidal Annular Plane Systolic Excursion.

Compared to T0, TAPSE was not significantly different at T1, but increased significantly at T2. RVO at T2 was significantly higher than at T0. There was no significant difference in RVO between T1 and T2. LVO at T2 was significantly higher than at T0. No difference in LVO between T0 and T1, or between T1 and T2 was documented.

#### TDI data

In all the infants myocardial velocities were higher in the right ventricle than in the left ventricle. In the left ventricle S’, E’ and A’ velocities, as well as the left ventricle E/E’ ratio, at T1 and T2 were not significantly different from those detected at T0. There was no significant difference in right S’ at 2 hours after surfactant administration; however, a significant increase was detected 24 hours after surfactant therapy. The right E’ and A’ velocities did not change significantly at 2 and 24 hours.

There was a significant correlation between left ventricle E and E’ at T2 (ρ *=* 0.6; *p* = 0.025), but not at T0 (ρ *=* 0.5; *p* = 0.06) and T1 (ρ *=* 0.25; *p* = 0.5).

Intra-observer reproducibility was low: for example, the median CV for TAPSE at T0 was 3.7% (interquartile range 0.92-8.9), while for left ventricle S’ velocity at T0 was 3.9% (interquartile range 3.4-8.3).

## Discussion

This pilot study is the first attempt to evaluate haemodynamic changes after surfactant administration using new echocardiographic techniques. We observed that, after the administration of surfactant, the AT/RVET ratio increased over time, and this increase became statistically significant at 24 hours compared to baseline. The increase in the AT/RVET was inversely correlated to pulmonary pressure, and the decrease in pulmonary pressure was associated with an increase in RVO, documented also after 2 hours. Previous studies have shown the change in PAP using Doppler measuring AT/RVET ratio in preterm after surfactant therapy. However, although the AT/RVET correlates with PAP, it can also be influenced by myocardial contractility [25].

The increase in RVO which followed the administration of surfactant could be due to a concomitant decrease in right ventricular afterload or to the maturation of the myocardial tissue after birth.

A significant increase in right peak systolic velocity and in TAPSE was documented at T2. Recently, Koestenberger *et al*. established normal reference values of TAPSE in preterm and term newborns [26]. In our study, data obtained in a more selected population of preterm infants with RDS confirmed that changes in TAPSE were in line with other parameters of right ventricular function. Thus, TAPSE can be considered as a reliable measure of right ventricular function also in preterm infants with nCPAP respiratory support in the first days of life and after surfactant therapy. These findings can be important for neonatal clinical practice, because TAPSE reproducibility is higher than that of other ECHO indices of right function. Furthermore, TAPSE is independent from heart rate variations [26].

With regard to LVO, no difference was documented in our patients at 2 hours compared to baseline; however, a significant increase in LVO was found after 24 hours from surfactant administration.

Previous data regarding the impact of surfactant administration on haemodynamics in preterm infants are conflicting. Some studies have shown that surfactant determined a fall in pulmonary pressure which was followed by an increase in transductal shunt [8], with a consequent reduction in RVO and an increase in LVO due to preload augmentation [4-6]. On the other side, Sehgal *et al.* studied the early consequences of surfactant administration in the delivery room, showing that giving surfactant within 30 minutes of birth was associated with an increase in the ductal size, a predominant left-to-right transductal flow, an increase in RVO and a decrease in LVO [10]. The authors hypothesized that this was due to an augmentation of right ventricular inflow via increased left-to-right transatrial flow, because the size of the *foramen ovale* is maximum immediately after birth. We can speculate that the lack of LVO increase observed in our patients at T1 could be due to the left-to-right flow across the *foramen ovale*. The significant increase of LVO at T2 could be explained instead by several reasons, including the changing flow across the *ductus arteriosus*, the adaptation of the left ventricle to the increase in afterload and the change in peripheral vascular resistance.

In the study by Sehgal *et al*., surfactant administration was followed by an early low diastolic arterial pressure [10]: the authors suggested that this could compromise coronary artery perfusion and lower cardiac output. In our study, TDI examination showed that both right and left myocardial velocities did not change after surfactant administration, except for right peak systolic velocity, which improved; thus, according to our data, it seems unlikely that surfactant can compromise myocardial perfusion.

TDI feasibility in preterm and term infants was recently demonstrated in some studies [12,16,17], but the applicability of TDI in neonatal clinical practice still needs to be defined. Similarly to Negrine *et al.*[15], our data, obtained in a more selected and homogeneous population, show that peak velocities in the right ventricle are higher than in the left ventricle.

This study presents several limitations: the number of included infants is small, and the range of gestational age and birth weight is quite wide; furthermore, a single operator performed all the scans, and it was not possible to calculate the interobserver variability, especially for TDI.

In conclusion, we observed that after surfactant administration TAPSE significantly increased over time in agreement with other parameters of right ventricular function such as AT/RVET and RVO; thus the use of TAPSE in this population appears to be reliable. Moreover, TDI data suggest that surfactant administration in preterm infants with RDS did not impair myocardial function. These observations encourage the development of further studies investigating the role of TDI and TAPSE in the neonatal population.

## Abbreviations

AT: Acceleration time; CHD: Congenital heart disease; ECHO: Echocardiography; LMA: Lateral mitral annulus; LTA: Lateral tricuspid annulus; LVO: Left ventricular output; NICU: Neonatal intensive care unit; PAP: Pulmonary artery pressure; PDA: Patent ductus arteriosus; PFO: Patent foramen ovale; PWD: Pulse wave doppler; RDS: Respiratory distress syndrome; RVET: Right ventricular ejection time; RVO: Right ventricular output; TAPSE: Tricuspidal annular plane systolic excursion; TDD: Transductal diameter; TDI: Tissue doppler imaging.

## Competing interests

None of the authors has any conflict of interest to declare in connection with this paper.

## Authors’ contributions

FV and GF designed the study. SG performed ECHO, supervised by MF and AB. GA and AA analysed and interpreted data. FV, SG and AA drafted the manuscript; the first draft was revised by MF, AB and GF. All the authors gave final approval of the version to be submitted.
